# The comparison of efficacy and complications of coblation and radiofrequency thermocoagulation for V2/V3 idiopathic trigeminal neuralgia: a retrospective cohort study of 292 cases

**DOI:** 10.1186/s12871-020-01224-2

**Published:** 2021-01-07

**Authors:** Chenhui Wang, Zhi Dou, Mengwei Yan, Yuanzhang Tang, Rui Zhao, Yujie Han, Jiaxiang Ni

**Affiliations:** 1 Department of Pain Management, Xuanwu Hospital, Capital Medical University, No.45 Changchun Street, Xicheng District, Beijing, 100053 China; 2 Capital Medical University, Fengtai District, Beijing, 100069 China

**Keywords:** Trigeminal neuralgia, Radiofrequency thermocoagulation, Coblation

## Abstract

**Background:**

Coblation is a novel technique in respect of treating idiopathic trigeminal neuralgia. We aimed to identify the efficacy and complications between radiofrequency thermocoagulation (RFT) and coblation for V2/V3 idiopathic trigeminal neuralgia (ITN) and investigate the risk factors associated with postoperative facial numbness.

Methods: We retrospectively reviewed our cohort of 292 patients who had undergone RFT or coblation for V2/V3 ITN. The characteristics of the baseline were collected before surgery. Pain scores, the degree of facial numbness and other complications were evaluated at discharge and 1 month, 3 months, 6 months and 12 months after surgery.

**Results:**

Postoperative pain intensity was apparently alleviated in both groups. The initial and 12-months remission rates were 94.0 and 75.3% in coblation group compared with 96.9 and 78.4% in RFT group (*P* = 0.462, *P* = 0.585). The degree of postoperative facial numbness tended to be more severe in RFT group at discharge, 1 month, 6 months and 12 months (*P* = 0.006, *P* = 0.026, *P* = 0.004, *P* = 0.003). Factors significantly associated with more severe facial numbness were procedure of RFT (OR = 0.46, 95%CI: 0.28–0.76, *P* = 0.002), history of previous RFT at the affected side (OR = 2.33, 95%CI: 1.21–4.48, *P* = 0.011), and ITN with concomitant continuous pain (OR = 0.36, 95%CI: 0.18–0.71, *P* = 0.004).

**Conclusion:**

Coblation could reduce the degree of postoperative facial numbness for ITN, and the efficacy was no less effective than RFT. History of previous RFT at the affected side, procedure of RFT, ITN with concomitant continuous pain was identified as significant factors of the development of postoperative facial numbness.

## Background

Trigeminal neuralgia (TN) is one of the most common types of neuropathic pain in the orofacial region. The annual morbidity of TN is approximately 5–28.6/100,000 [1–3], which is relatively higher among the elderly. It is a chronic pain syndrome characterized by recurrent paroxysmal, lightning-like, or acupuncture-like pain in the facial distribution along regions of trigeminal nerve [4]. However, the etiology and pathogenesis of TN have not been clearly defined at present. Initial treatment is generally based on a pharmacological approach. But lots of patients do not respond to drugs and also cannot endure the side effects of drug tolerance, such as nausea, vomiting, and dizziness [5, 6]. For these reasons, neurosurgery treatments, such as micro-vascular decompression [7] (MVD), Gasserian ganglion percutaneous techniques [8, 9], and stereotactic radiosurgery [10], need to be considered to relieve the pain. However, people are more likely to accept minimally invasive treatment like radiofrequency thermocoagulation (RFT), which has been widely used in the elderly who suffer from facial pain of trigeminal neuralgia, rather than the surgery that requires craniotomy and tracheal intubation. Although the remission rate of RFT is satisfactory at a 1-year follow-up, the recurrence rate of RFT ranges from 15 to 46% [11, 12] during long-term follow-up, with a multitude of patients complained about the facial numbness after RFT. Coblation is a novel technique, which was widely used in intraoperative tissue cutting of otolaryngology [13] and other fields. It depends on the plasma field of sodium ions generated by plasma knife head and normal saline, which can result in molecular dissociation [14, 15], rather than thermal damage. Currently, coblation has been reported in the treatment of thoracic neuralgia [16] and idiopathic trigeminal neuralgia [17, 18], while our previous small sample study and short-term follow-up results suggested that the analgesic effect of the coblation group at 3 months was better than that of the RFT group, and the incidence of facial numbness was even less in coblation group [19]. However, some issues of coblation about changes in facial numbness, the degree of numbness compared with patients after RFT, and the risk factors associated with the degree of numbness have not been cleared in our previous study. Therefore, our study aims to investigate long-term outcomes of efficacy and complications of coblation for V2/V3 ITN and to estimate risk factors associated with the degree of postoperative facial numbness.

## Methods

### Study design and patients

In this retrospective study, we identified all patients who underwent RFT or coblation for treatment of TN between August 2017 and April 2019 in the department of pain management at Xuanwu Hospital of Capital Medical University in China. Data collection and analysis was also approved by Institutional Review Board (IRB) of Xuanwu hospital. Written informed consent from patients was obtained before follow-up entry.

The inclusion criteria were as follows: (1) ITN of maxillary and/or mandibular divisions in accordance with the International Classification of Headache Disorders [20], (2) no space occupying lesions, (3) poor effect of drug therapy or side effects were intolerable.

The following patients were excluded: (1) patients with severe systemic diseases who were intolerable to surgery and anesthesia, (2) coagulation dysfunction or local infection in the puncture area before surgery, (3) patients with mental illness, who were unable to express subjective feelings clearly.

Clinical data of patients were extracted from the electronic medical records. All the patients who had undergone RFT or coblation for ITN were followed as the routine practice. The follow-up data were registered in the hospital data system. Each patient was taken physical examination to assess pain, numbness and other complications at discharge (3 days after surgery). Long term follow-up was carried out at 1 month, 3 months, 6 months and 12 months by telephone interview by specially trained investigators on schedule. Although telephone interview carries risks of bias including the characteristics of the respondent and the examiner, it was acceptable for patients who were unable to come to our center for further examination.

### Grouping

The patients were grouped according to the procedures they selected after an informed comprehensive discussion with their surgeon: RFT group and coblation group. All procedures were performed in an operating room under fluoroscopic guidance of C-arm.

### RFT group

Vital signs were monitored in operating room. The patient was lying in supine position with the head keeping overhanging. After the visualization of foramen ovale (FO) by C-arm, the patients underwent a sterile preparation and local anesthesia. Then a 22-G puncture needle (15 cm, with a 5-mm active tip; Cosman TICC5 electrode, Cosman Medical, Burlington, MA, U.S.A.) was advanced into the FO using the Hartel anterior approach. C-arm was then performed to confirm the location of the needle tip and the tip was adjusted according to patients’ reactions to the sensory (50 Hz, 1 ms) and motor (2 Hz, 0.1 ms) stimulation test until paresthesia was elicited in the affected area. After positioning was accomplished satisfactorily, patients were administered intravenous anesthesia (propofol 1.5–2 mg/kg). Thermocoagulation was performed at 70 °C for 180 s.

### Coblation group

The procedures before verifying the position of the needle tip were the same as the RFT group. When the needle was located in FO, we connected the anode of the neuro-stimulator (B. Braun Co., Ltd., Melsungen, Germany) to the tail of coblation needle (DXR-G1100-A185; Xi’an Surgical Medical Technology Co., Ltd., Xi’an, China) and the cathode was connected with patient’s skin. We turned on the neuro-stimulator and adjusted it to 2 Hz and 0.1 ms, and gradually increased the intensity of the stimulus until it caused reproducible pain at 0.5 mV. When reproducible pain was successfully repeated, intravenous anesthesia was applied (propofol 1.5–2 mg/kg). The ablation energy and the coagulation energy of low-temperature plasma multifunction operating system (SM-D380C; Xi’an Surgical Medical Technology Co., Ltd., Xi’an, China) was level 1 and parameters were unchanged during the operation. The ablation time was 30 s, and the ablation was performed again after 30 s.

### Clinical materials

Clinical characteristics of patients were collected from electronic medical records, including gender, age, disease duration, affected side (left, right), co-morbidity (diabetes mellitus or not), baseline pain numeric rating scale (NRS) scores (0 = no pain, 10 = severe pain), pain characteristics (paroxysmal, continuous), pain distribution in anatomic trigeminal nerve dermatome (V2, V3, V2 + V3), and history of previous treatment at the affected side (none, MVD, RFT, other).

All patients were followed up by telephone interviews at 1-month, 3-months, 6-months and 12-months by our investigators respectively. Follow-up data included NRS scores of pain intensity, pain relief, the degree of postoperative facial numbness, recovery from postoperative facial numbness and other complications.

Pain intensity was assessed using NRS score (0 = no pain, 10 = severe pain). Pain relief was considered when the NRS score was less than 4. For facial numbness, the degree of facial numbness was assessed by the Barrow Neurological Institute (BNI) facial hypesthesia scale [21]: Class I: no facial numbness; Class II: mild facial numbness and not bothersome; Class III: facial numbness and somewhat bothersome; Class IV: facial numbness and very bothersome. With regard to postoperative facial numbness, we observed three outcomes at the endpoint of our study: (1) no numbness: patients without subjective facial numbness; (2) remission: the degree of facial numbness was improved by 1–2 BNI level but still hadn’t reached BNI I; (3) no remission: postoperative facial numbness was not changed or even raised some levels. Besides, none of the patients had subjective facial numbness before surgery and all the patients had stopped taking medicines before surgery. Other complications such as oral ulcer, corneal hypoesthesia, masticatory weakness, hypoesthesia of temperature and tinnitus were recorded as well.

### Statistical analysis

Under normal distribution and equality of variances, quantitative data were reported as means ± standard deviation (SD) and were compared using the independent-sample t test or the paired-sample t test. Otherwise, non-normal variables were reported in the median with interquartile range (IQR) and Wilcoxon rank-sum test was used. Categorical data were described by frequencies and proportions and were compared by Pearson’s chi-square test or the Wilcoxon rank-sum test.

Univariate and multivariate ordered logistic regression analysis was used to assess the statistical significance of the potential variables in patients’ clinical characteristics, with previous parallel lines test (likelihood ratio test). Each relevant parameter was calculated with an associated confidence interval (CI) and odds ratios (ORs) respectively.

Statistical analyses were performed using SPSS software, version 23.0 (IBM SPSS, New York, NY, USA). The level of significance was set at *P* < 0.05 (2-tailed).

## Results

### Characteristics of the patients

A total of 292 patients underwent RFT or Coblation procedure; however, 25 patients in RFT group and 20 patients in coblation group were lost to follow-up. Eventually, we obtained complete follow-up data of 247 cases, including 97 patients in RFT group and 150 patients in coblation group.

The baseline characteristics of the patients are summarized in Table 1. There were no statistically significant differences in age, sex, affected side, the distribution of pain, disease duration, pain characteristics, co-morbidity, history of previous treatment at affected side and preoperative NRS scores between 2 groups.

**Table 1:** Demographic characteristics of patients

Variables	RFT (*n* = 97)	Coblation (*n* = 150)	*P*
Age (mean ± SD)(y)	63.51 ± 11.46	61.07 ± 10.44	0.086
Sex, n (%)			0.716
Male	34 (35.1%)	56 (37.3%)	
Female	63 (64.9%)	94 (62.7%)	
Affected side, n(%)			0.055
Left	29 (29.9%)	63 (42%)	
Right	68 (70.1%)	87 (58%)	
Distribution of pain, n(%)			0.065
V2	24 (24.7%)	30 (20%)	
V3	18 (18.6%)	48 (32%)	
V2 + V3	55 (56.7%)	72 (48%)	
Disease duration (mean ± SD) (mo)	64.75 ± 64.98	66.87 ± 75.09	0.820
Pain characteristics, n(%)			0.846
Paroxysmal	83 (85.6%)	127 (84.7%)	
Continuous	14 (14.4%)	23 (15.3%)	
Co-morbidity, n(%)			0.432
Diabetes mellitus	8 (8.2%)	17 (11.3%)	
No diabetes mellitus	89 (91.8%)	133 (88.7%)	
History of previous treatment at the affected side, n(%)			0.499
Previous RFT	18 (18.6%)	25 (16.7%)	
Previous MVD	8 (8.2%)	6 (4%)	
Other	4 (4.1%)	8 (5.3%)	
None	67 (69.1%)	111 (74%)	

### Efficacy

We started our postoperative follow-up at discharge (3 days after surgery). NRS scores were significantly lower in both groups at discharge compared with pre-surgery (RFT: *t* = 37.194, *P* < 0.001; Coblation: *t* = 44.949, *P* < 0.001). There had been no cases of anesthesia dolorosa at discharge. There was no significant difference in the mean NRS scores between the two groups at discharge, 1-month, 3-months, 6-months and 12-months of follow-up (*P* > 0.05). Postoperative pain intensity is shown in Table 2.

**Table 2:** Different time of NRS scores between 2 groups

NRS score (mean ± SD)	RFT (*n* = 97)	Coblation (*n* = 150)	t	95%CI of the Difference	*P*
Baseline	7.65 ± 1.35	7.72 ± 1.31	−0.407	−0.414-0.270	0.684
Discharge	1.23 ± 1.10	1.17 ± 1.27	0.383	−0.249-0.369	0.702
1 month	0.96 ± 1.77	0.93 ± 1.84	0.108	−0.440-0.491	0.914
3 months	1.12 ± 1.99	1.23 ± 2.15	−0.379	−0.638-0.433	0.705
6 months	1.19 ± 2.11	1.42 ± 2.30	−0.808	−0.806-0.337	0.420
12 months	1.58 ± 2.51	1.71 ± 2.45	−0.401	−0.765-0.506	0.689

NRS score is considered as quantitative data and independent-sample t test is used to assess it

*CI* Confidence interval

When the postoperative NRS score was < 4, pain relief was considered. At discharge, the remission rate (pain relief) was 96.9% (94/97) in RFT group, whereas it was 94.0% (141/150) in coblation group (correction for continuity of χ^2^ = 0.540, *P* = 0.462). At 1 month after surgery, the remission rate was 90.7% (88/97) in RFT group and 89.3% (134/150) in coblation group (χ^2^ = 0.125, *P* = 0.724). At 3 months after surgery, the remission rate was 86.6% (84/97) in RFT group and 84.0% (126/150) in coblation group (χ^2^ = 0.312, *P* = 0.576). At 6 months after surgery, the remission rate was 85.6% (83/97) in RFT group and 82.0% (123/150) in coblation group (χ^2^ = 0.541, *P* = 0.462). At 12 months after surgery, the remission rate was 78.4% (76/97) in RFT group and 75.3% (113/150) in coblation group (χ^2^ = 0.298, *P* = 0.585).

### Complications

Facial numbness on the affected side was the most frequent complication after surgery. The incidence of postoperative facial numbness in the RFT group at discharge was 91.8% (89/97), and the proportions of each grade were: BNI I 8.2% (8/97), BNI II 23.7% (23/97), BNI III 60.8% (59/97), BNI IV 7.2% (7/97). However, 88% (132/150) of the patients in coblation group had postoperative facial numbness at discharge, and the proportions of each grade were: BNI I 12% (18/150), BNI II 36% (54/150), BNI III 50.7% (76/150), BNI IV 1.3% (2/150). At the end point of our follow up, we found that in RFT group, the degree of postoperative facial numbness was alleviated in 36.1% of the patients (35/97), 19.5% (19/97) patients had no subjectively facial numbness and 44.3% (43/97) patients remained unalleviated. In coblation group, postoperative facial numbness was gradually alleviated in 25.3% of the patients (38/150), 34.7% (52/150) patients had no subjectively facial numbness and 40% (60/150) patients remained unalleviated. In addition, the proportions of the two groups were: RFT group: BNI I 19.6% (19/97), BNI II 43.3% (42/97), BNI III 35.1% (32/97) BNI IV 2.1% (2/97); Coblation group: BNI I 34.7% (52/150), BNI II 42.7% (64/150), BNI III 21.3% (32/150), BNI IV 1.3% (2/150). Complications after different procedures are recorded in Tables 3 and 5. Changes in facial numbness after surgery are presented in Table 4.

**Table 3:** Complications except for facial numbness after surgery

Variables	RFT	Coblation	*χ*^*2*^	*P*
Oral ulcer, n(%)	5 (5.2%)	10 (6.7%)	0.236	0.627
Masticatory weakness, n(%)	23 (23.7%)	40 (26.7%)	0.271	0.603
Corneal hypoesthesia, n(%)	21 (21.6%)	36 (24%)	0.183	0.669
Hypoesthesia of temperature, n(%)	30 (30.9%)	51 (34%)	0.252	0.615
Tinnitus, n(%)	14 (14.4%)	26 (17.3%)	0.365	0.546

Complication is considered as categorical data and Pearson’s chi-square test is used to assess it

**Table 4:** The recovery state from postoperative facial numbness at 12 months

	No numbness, n(%)	Remission, n(%)	No remission, n(%)	Z	*P*
RFT (*n* = 97)	19 (19.5%)	35 (36.1%)	43 (44.3%)	2.828	0.093
Coblation (*n* = 150)	52 (34.7%)	38 (25.3%)	60 (40.0%)		

The recovery state is considered as ranked data and nonparametric test (Independent-Samples Kruskal-Wallis Test) is applied to assess it

According to the results showed in Tables 3, 4 and 5, there was a significant difference between two groups in the degree of facial numbness except for 3-months and the mean-rank of two groups suggested the degree of postoperative facial numbness tended to more severe in RFT group. However, there was no significant difference in the prognosis of facial numbness between the two groups although some advantage was showed in the rate of “no numbness” in coblation group.

**Table 5:** The postoperative facial numbness (BNI I-IV) in follow up

	Discharge		1 month		3 months		6 months		12 months	
	RFT (*n* = 97)	Coblation (*n* = 150)	RFT (*n* = 97)	Coblation (*n* = 150)	RFT (*n* = 97)	Coblation (*n* = 150)	RFT (*n* = 97)	Coblation (*n* = 150)	RFT (*n* = 97)	Coblation (*n* = 150)
I, n (%)	8 (8.2%)	18 (12%)	9 (9.3%)	20 (13.3%)	12 (12.4%)	29 (19.3%)	15 (15.5%)	40 (26.7%)	19 (19.6%)	52 (34.7%)
II, n (%)	23 (23.7%)	54 (36%)	25 (25.8%)	54 (36%)	34 (35.1%)	59 (39.3%)	41 (42.3%)	71 (47.3%)	42 (43.3%)	64 (42.7%)
III, n (%)	59 (60.8%)	76 (50.7%)	59 (60.8%)	73 (48.7%)	48 (49.5%)	59 (39.3%)	38 (39.2%)	37 (24.7%)	32 (35.1%)	32 (21.3%)
IV, n (%)	7 (7.2%)	2 (1.3%)	4 (4.1%)	3 (2.0%)	3 (3.1%)	3 (2.0%)	3 (3.1%)	2 (1.3%)	2 (2.1%)	2 (1.3%)
Mean-Rank	138.07	114.9	135.38	116.64	134.04	117.51	139.35	114.08	139.80	113.78
Median	III	III	III	III	III	II	II	II	II	II
Wilcoxon W	17,235.0		17,496.5		17,626.5		17,111.5		17,067.0	
*P*	0.006^*^		0.026^*^		0.056		0.004^*^		0.003^*^	

BNI is considered as ranked data and Wilcoxon rank-sum test was used to assess it

^*^P < 0.05

Other complications such as oral ulcer, masticatory weakness, corneal hypoesthesia, hypoesthesia of temperature and tinnitus were not significantly different between 2 groups (*P* > 0.05).

### Risk factors associated with postoperative facial numbness for ITN

The degree of numbness (BNI I, BNI II, BNI III-IV) was taken as the dependent variable. As a small proportion of BNI IV could not ensure the accuracy of the model, we combined it with BNI III into BNI III-IV, representing moderate or severe postoperative facial numbness. Firstly, we performed a univariate ordered logistic regression analysis between the outcomes and each independent variable of patients’ characteristics. Variables with *P* < 0.05 were likely to entered subsequent multivariate analysis. Subsequently, we constructed a multivariate ordered logistic regression model using the enter procedure among the potential candidate variables. Variables were examined for multicollinearity before entering regression model and there was no multicollinearity between the independent variables.

Table 6 presents the potential variables related to the degree of postoperative facial numbness, and the results of univariate analysis. Based on the results of univariate analysis, four of the variables (disease duration, pain characteristic, history of previous treatment at the affected side, procedure) were considered to be candidate variables. These variables were then used for multivariate ordered logistic regression analysis. Factors significantly associated with more severe facial numbness were procedure of RFT (OR = 0.46, 95%CI: 0.28–0.76, *P* = 0.002), history of previous RFT at the affected side (OR = 2.33, 95%CI: 1.21–4.48, *P* = 0.011), and ITN with concomitant continuous pain (OR = 0.36, 95%CI: 0.18–0.71, *P* = 0.004), which was presented in Table 7.

**Table 6:** Univariate ordered logistic regression analysis for the factors associated with the degree of postoperative numbness at 12-months

Variables	BNI I	BNI II	BNI III-IV	LR-test	*P*	OR	95% Confidence Interval
Age (years) (mean ± SD)	61.7 ± 13.1	61.5 ± 10.4	63.1 ± 9.1	0.47	0.416	1.01	0.99–1.03
Sex, n (%)				0.52			
Male	23 (25.6%)	41 (45.6%)	26 (28.9%)		0.558	1.15	0.71–1.59
Female	48 (30.6%)	65 (41.4%)	44 (28.0%)				
Affected side, n (%)				0.68			
Right	44 (28.4%)	65 (41.9%)	46 (29.7%)		0.649	0.89	0.55–1.46
Left	27 (29.3%)	41 (44.6%)	24 (26.1%)				
Distribution of pain, n (%)				0.59			
V2	17 (31.5%)	26 (48.1%)	11 (20.4%)				
V3	22 (33.3%)	26 (39.4%)	18 (27.3%)		0.745	1.12	0.57–2.18
V2 + V3	32 (25.2%)	54 (42.5%)	41 (32.3%)		0.147	1.55	0.86–2.11
Disease duration (months) (mean ± SD)	58.1 ± 66.4	61.8 ± 60.0	80.5 ± 89.2	0.56	0.048^*^	1.00	1.00–1.01
Pain characteristic				0.46			
Paroxysmal	65 (31.0%)	94 (44.8%)	51 (24.3%)		0.001^*^	0.33	0.17–0.65
Continuous	6 (16.2%)	12 (32.4%)	19 (51.4%)				
Co-morbidity, n (%)				0.76			
Diabetes mellitus	8 (32%)	10 (40%)	7 (28%)		0.798	0.90	0.42–1.95
No diabetes mellitus	63 (28.4%)	96 (43.2%)	63 (28.4%)				
History of previous procedure at the affected side, n (%)				0.46			
Previous RFT	9 (20.9%)	13 (30.2%)	21 (48.8%)		0.002^*^	2.79	1.47–5.26
Previous MVD	2 (14.3%)	7 (50%)	5 (35.7%)		0.137	2.17	0.78–6.02
Other	2 (16.7%)	5 (41.7%)	5 (41.7%)		0.112	2.44	0.81–7.34
None	58 (32.6%)	81 (45.5%)	39 (21.9%)				
Treatment, n (%)				0.81			
RFT	19 (19.6%)	42 (43.3%)	36 (37.1%)				
Coblation	52 (34.7%)	64 (42.7%)	34 (22.7%)		0.003^*^	0.48	0.30–0.78
Baseline NRS score (mean ± SD)	7.68 ± 1.36	7.81 ± 1.32	7.53 ± 1.29	0.22	0.513	0.94	0.79–1.12
NRS score at discharge (mean ± SD)	1.15 ± 1.01	1.08 ± 1.07	1.39 ± 1.53	0.28	0.235	1.12	0.93–1.36

*LR test* Likelihood ratio test, *OR* Odds ratio

^*^ *P* < 0.05

**Table 7:** Ordered logistic regression analysis results for variables by the enter procedure

Variables	LR-test	*P*	OR	95% Confidence Interval
Disease duration (months) (mean ± SD)	0.755	0.188	1.00	0.99–1.01
Pain characteristic				
Paroxysmal		0.004^*^	0.36	0.18–0.71
Continuous				
History of previous neurosurgery, n (%)				
Previous RFT		0.011^*^	2.33	1.21–4.48
Previous MVD		0.526	1.41	0.49–4.11
Other		0.181	2.16	0.70–6.48
None				
Treatment, n (%)				
RFT				
Coblation		0.002^*^	0.46	0.28–0.76

Test of Parallel Lines (LR test, Chi-Square = 3.415, *P* = 0.755)

*LR test* Likelihood ratio test, *OR* Odds ratio

^*^ *P* < 0.05

## Discussion

Coblation is a relatively novel technique applied in neuropathic pain. Some previous studies found [19] that the coblation group had lower postoperative NRS scores than RFT group and the risk of postoperative numbness in patients with V1, V2, and V3 ITN was reduced. However, the previous studies were limited to small sample sizes and short-term follow up. Besides, because of the specificity of V1, some studies indicated that the appropriate temperature of RFT for the treatment of V1 ITN was lower than V2/V3 ITN [22, 23], which was to avoid complications such as diplopia and keratitis. To prevent the influences of different temperatures on the evaluation of surgical outcomes, we only included V2/V3 ITN in the present study with a 12-months follow-up.

In terms of the efficacy of coblation, the postoperative NRS scores at discharge were significantly lower than before surgery and it continuously remained at a low level during our 12-months follow up, which was identical with RFT group in respect of postoperative analgesic effect. Compared with the initial remission rate of 96.9% in RFT group, it was 94% in coblation group, which was consistent with the result of a previous study ranging from 85.3 to 97.7% [19]. At the endpoint of follow up, the remission rate was 75.3 and 78.4%, respectively in coblation group and RFT group. Despite the slightly lower remission rate in coblation group, there was no statistically significant difference. Some literature review reported that the remission rate after RFT [22, 24] ranged from 50.4 to 80.7% in a follow-up of 5 years. However, as a relatively novel technique for the treatment of ITN, it was difficult to perform the long-term results according to the available and limited clinical data. Therefore, a big-sample of long-term follow-up study is still required to illustrate the efficacy of coblation.

Concerning postoperative complications, postoperative facial numbness had been the most disturbing issue among patients who accepted minimally invasive treatment of ITN. In contrast to the remission rate, the coblation group had some advantages in the degree of postoperative facial numbness compared with the RFT group. Our study results indicated that the degree of postoperative facial numbness tended to increase in RFT group, mainly due to the different mechanisms of the two techniques. Coblation [14, 15] is a unique modality that uses bipolar radiofrequency energy to ablate and coagulate soft tissue at low temperatures (40 °C–70 °C) with minimal thermal damage to surrounding tissues. During coblation, conductive saline solution is converted in the gap between the needle tip and the tissue and it is excited into an ionized plasma layer. Once the plasma layer meets the tissue, intercellular bonds would be destroyed by the ions. However, the mechanism of RFT is thermal damage as Aδ and C-type nerve fibers for pain transmission would be coagulated and denatured by temperatures of ≤80 °C, but Aα and Aβ nerve fibers [25, 26] would not be affected. Yao [22, 23] and Tang [9] had investigated the eligible temperature of RFT for V2/V3 ITN and the recommended temperature was 68–75 °C, which could reduce the incidence of postoperative facial numbness, but the temperature was still relatively high compared with coblation. Neither of the two procedures could selectively destroy C-type nerve fibers. But thermal damage to the nerves is minimized by coblation, which attributes to 40 °C–70 °C operating temperatures. This may be explanations of the fact that lower degree of postoperative facial numbness occurred in coblation group rather than RFT group. For the non-significant outcome of numbness at 3 months, the possible reason might be information bias existed in this study - the follow-up data might be influenced by different mood of the examiner and possible suggestibility of the examiner. Other complications such as oral ulcer, masticatory weakness, corneal hypoesthesia, hypoesthesia of temperature and tinnitus were no significant differences between the 2 groups.

Compared with the preferred 70 °C 60s RFT with 2 mm active tips, we adopted 70 °C 180 s RFT with 5 mm active tips in our center because good pain relief, less recurrence rate and less complications can be obtained under this kind of condition, based on published literature [22, 23]. Theoretically, ablation temperature, ablation time and radius of active tips have impact on numbness. However, in terms of most studies about RFT (70 °C 180 s with 5 mm active tips) for ITN and our study, RFT procedures have much numbness than coblation.

In addition, complications are also important aspects in evaluating the effectiveness of treatment and improving the satisfaction of patients. However, due to the mechanism of treatment, postoperative facial numbness was almost inevitable among patients who accepted minimally invasive treatment. To explore the risk factors of the degree of postoperative facial numbness in ITN patients, we conducted a multivariate analysis and the results showed that the pain characteristics, previous treatment at the affected side, and procedure were risk factors significantly associated with the degree of postoperative facial numbness. The continuous pain [27, 28] was described as dull, aching, burning and other different from the sharp-like. Some studies reported [27] that the prognosis of continuous ITN patients was less effective than paroxysmal ITN patients, with 80% initial remission rate and 54% 5-years remission rate. However, we also observed more severe postoperative facial numbness in patients with continuous ITN in our study, which might be explained by the central sensitization hypothesis [29]. The hypotheses rated that the intense, repetitive and sustained stimulus could increase the membrane excitability and synaptic efficacy, and decrease the thresholds of activation. The chronic and continuous stimulus to trigeminal nerve would lead to pain hypersensitivity, that is, innocuous stimulation could be considered as pain-causing noxious stimulation. We speculated that the pain hypersensitivity caused by pathophysiological changes in pain pathway would also affect the superficial sensation, which would be the possible cause of the severe postoperative facial numbness. In addition, some scholars also rated [28] that the continuous pain was transformed from paroxysmal pain over time if left untreated, and the changes involved the development of sensory impairment. But it is just supposition; evidence is needed to support the speculation.

Meanwhile, we also found that patients with recurrent ITN, who had accepted RFT, suffered more severe facial numbness compared with primary ITN patients. The destruction of the Gasserian ganglion by neurosurgeries would result in denaturation and necrosis of partial trigeminal nerve, and the damaged nerve fibers would be gradually repaired physiologically, which may lead to the recurrence of ITN [30]. And we supposed that the second damage to the repaired nerves would result in severe facial numbness, compared with those who accepted primary destructive neurosurgeries. But more researches are needed to support our supposition.

There are still some limitations about our study. First, patients with V1 ITN were excluded because of the appropriate ablation temperature differed from V2/V3 ITN. Second, it was a retrospective study and selection bias could not be avoided. Large-scale, randomized, double-blind studies will be needed to validate our findings in the future.

## Conclusion

Coblation is an effective treatment for V2/V3 ITN. It could reduce the degree of postoperative facial numbness for ITN, and the efficacy is no less effective than RFT. History of previous RFT at the affected side, procedure of RFT, ITN with concomitant continuous pain was identified as significant factors of the development of postoperative facial numbness. These findings would be considered to improve the satisfaction of patients after surgery during our clinical work.

## Data Availability

The datasets used and analyzed during the current study are available from the corresponding author on reasonable request.

## Abbreviations

RFT: Radiofrequency thermocoagulation
ITN: Idiopathic trigeminal neuralgia
TN: Trigeminal neuralgia
MVD: Micro-vascular decompression
IRB: Institutional review board
FO: Foramen ovale
NRS: Numerical rating scale
BNI: Barrow neurological institute
SD: Standard deviation
IQR: Interquartile range
CI: Confidence interval
OR: Odds ratio
